# A nuclear role for Atg8-family proteins

**DOI:** 10.1080/15548627.2020.1794356

**Published:** 2020-07-18

**Authors:** Anne-Claire Jacomin, Stavroula Petridi, Marisa Di Monaco, Ioannis P. Nezis

**Affiliations:** School of Life Sciences, University of Warwick, Coventry, UK

**Keywords:** Acetylation, autophagy, LIR motif, nucleus, transcription, LC3/Atg8

## Abstract

Despite the growing evidence that the macroautophagy/autophagy-related protein LC3 is localized in the nucleus, why and how it is targeted to the nucleus are poorly understood. In our recent study, we found that transcription factor seq (sequoia) interacts via its LIR motif with Atg8a, the *Drosophila* homolog of LC3, to negatively regulate the transcription of autophagy genes. Atg8a was found to also interact with the nuclear acetyltransferase complex subunit YL-1 and deacetylase Sirt2. Modulation of the acetylation status of Atg8a by YL-1 and Sirt2 affects the interaction between seq and Atg8a, and controls the induction of autophagy. Our work revealed a novel nuclear role for Atg8a, which is linked with the transcriptional regulation of autophagy genes.

Macroautophagy is a type of autophagy where double-membrane compartments – phagophores – are being formed to engulf part of the cytoplasm, enclosing it within an autophagosome and targeting it for lysosomal degradation. Atg8-family proteins play an essential role in the regulation of autophagy as they enable the formation of the autophagosomes, as well as mediate the degradation of cargoes. As basal-level autophagy is very low, an efficient mechanism is required to induce autophagy in organisms that are put under stress by extracellular cues, such as nutrient deprivation. Although there is a body of evidence that indicates that Atg8-family proteins are enriched in the nucleus in nutrient-replete conditions, the machinery by which they are targeted to the nucleus, and thus the nuclear components with which they interact, remain largely uncharacterized. Uncovering these components may therefore be key in advancing our understanding of how autophagy-related proteins govern cellular responses to starvation.

A hallmark of most Atg8-interacting proteins is the presence of an LC3-interacting region (LIR) motif, which is required for their interaction with the Atg8-family proteins. The requirement for LIR motifs to convey the ability of autophagy-related proteins to interact with Atg8-family members is conserved across eukaryotes. The *Drosophila* Atg8a protein interacts with autophagy machinery components and selective autophagy receptors, through a LIR motif. We used an *in silico* approach for the identification of putative nuclear Atg8-family protein interactors based on a predicted LIR motif. This resulted in the identification of putative LIR motif-containing proteins susceptible to interact with Atg8a and to be involved in acetylation modulation: the transcription regulator seq (sequoia) and a subunit of the NuA4-Tip60 acetyltransferase complex, YL-1 [1].

The direct interaction between these proteins and Atg8a was validated, with a LIR motif-dependent interaction between seq and Atg8a confirmed. Moreover, we showed that the lack of *seq* in *Drosophila* larvae fat body cells – a larval tissue well-known for its ability to activate autophagy upon starvation – leads to the induction of autophagy through the generation of autophagosomes and their fusion with lysosomes [1]. This exceptional effect is observed notably in nutrient-rich conditions when autophagy activity is low and only maintained to the basal level. Our data support previous findings about Rph1 and KDM4A, yeast and mammalian homologs of seq, and the negative transcriptional regulation of autophagy.

Most LIR motif-containing proteins interact with Atg8-family proteins in the cytoplasm and relate to the formation of autophagosomes or to the recruitment of cargoes for degradation. However, seq is maintained in the nucleus upon starvation, suggesting that the interaction between seq and Atg8a orchestrates the regulation of autophagy induction exclusively from this organelle.

seq is a transcription factor, and its involvement in the repression of autophagy was uncovered when it was observed to bind to the promoter regions of autophagy genes in fed conditions, thereby reducing their transcription. This is exemplified by an observed induction in the expression of autophagy genes in *seq*-depleted larvae. Further to this, the LIR motif-deficient form of seq, which exhibits a reduced ability to bind to Atg8a, is less enriched at the promotor region of autophagy genes, which correlates to their upregulation when compared to wild-type seq. Interestingly, we observed that the absence of Atg8a has a significant negative impact on the expression of the same set of autophagy genes during starvation; however, a lack of Atg9 (a non-nuclear protein, which is part of a different complex required for the initiation of autophagosome formation) demonstrates no impact on their expression. This highlights the transcriptional importance of the nuclear interaction between Atg8a and seq in the expression of autophagy genes in starvation conditions. This is further emphasized in the observed association of Atg8a with the promoter region of autophagy genes, which is assumed to be by virtue of its interaction with seq [1].

The interaction between seq and Atg8a may contribute to the sequestration of Atg8a in the nucleus. Hence, in the absence of an interaction between Atg8a and seq (as showcased in the LIR mutant), the “releasing” of Atg8a by seq and its subsequent translocation into the cytoplasm, may also play a key role in lifting seq off the promotor region of autophagy genes; thereby elevating the repressive cap enforced upon these regions and initiating the induction of autophagy (Figure 1).

**Figure 1.**
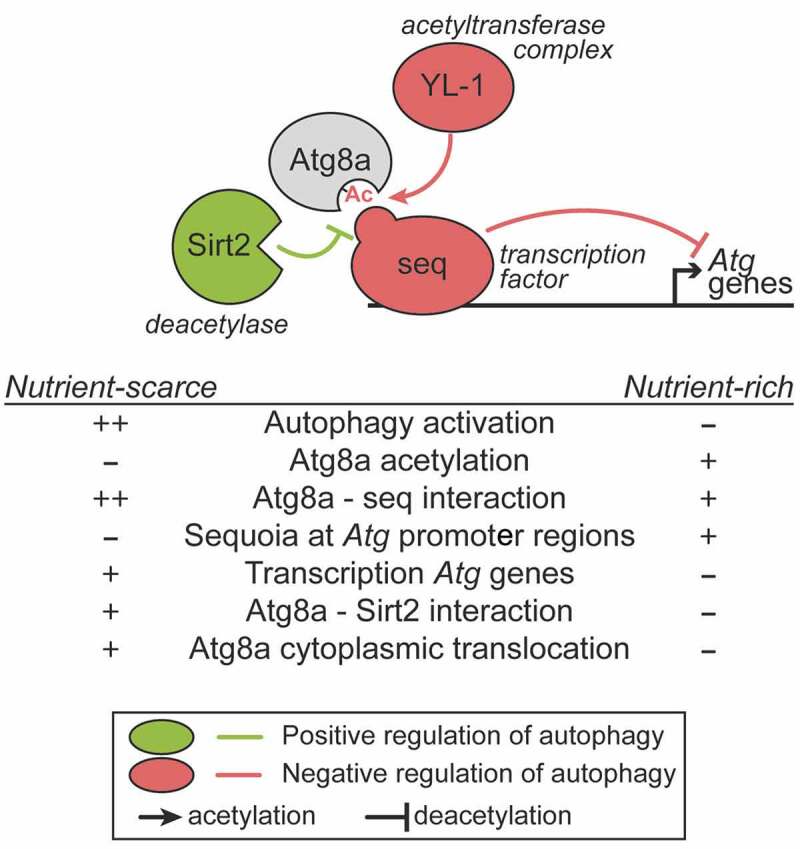
Role of Atg8a and seq in the negative regulation of autophagy. In nutrient-rich conditions, the transcription factor seq interacts with Atg8a in a LIR motif-dependent manner, and localizes at the promoter regions of autophagy-related (*Atg*) genes to repress their expression. In such conditions, Atg8a acetylation is maintained by its interaction with an acetyltransferase complex via YL-1. When nutrients are scarce, Atg8a is deacetylated by Sirt2, resulting in a stronger binding to seq, which can be lifted from the autophagy gene promoters, leading to their transcriptional activation.

In mammalian cells, nuclear LC3 protein is acetylated in nutrient-rich conditions, and its deacetylation is associated with its ability to translocate from the nucleus to the cytoplasm. We showed that *Drosophila* Atg8a is also acetylated and that starvation induces its deacetylation. We found that the YL-1 and the deacetylase Sirt2 are involved in the modulation of the acetylated status of Atg8a [1]. YL-1 protein is a subunit of a nuclear acetyltransferase complex known as SWR1 in yeast and the related SRCAP and NuA4-KAT5/Tip60 complexes in mammals, that control histone acetylation. We showed that the interaction between Atg8a and YL-1 is direct and does not depend on a functional LIR motif. Depletion of YL-1 by using an RNAi line, results in a reduced level of acetylated Atg8a in both fed and starved conditions. We showed that the acetylation status of Atg8a governed by YL-1 contributes to the modulation of the interaction between seq and Atg8a in the nucleus. Therefore, our data suggest that YL-1 is a negative regulator of autophagy, acting to maintain the acetylated status of Atg8a and its nuclear localization [1].

Acetylation of Atg8a by YL-1 is counter-balanced by the deacetylase Sirt2, which is the *Drosophila* homolog of the mammalian SIRT1 (sirtuin 1). The lack of autophagy induction in *Drosophila* larvae fat body cells upon starvation that we observed in Sirt2 mutants, confirms previous reports that this deacetylase is a positive regulator of autophagy. We showed that Sirt2 mutants exhibit reduced Atg8a lipidation during starvation, whereas overexpression of Sirt2 results in increased lipidation of Atg8a, both in fed and starved conditions. Furthermore, we showed that Sirt2 overexpression leads to decreased acetylation of Atg8a both in fed and starved conditions and that Sirt2 preferentially interacts with Atg8a when larvae are starved. This observation suggests that the binding of Sirt2 to Atg8a may contribute to Atg8a deacetylation and export to the cytoplasm. Furthermore, we suggest that in the event of deacetylation of Atg8a, due to its conformational change, its interaction with seq strengthens. This thus implies that deacetylation by Sirt2 may have a crucial role in the ability of Atg8a to lift seq off the promotor region of autophagy-related genes in nutrient-scarce conditions [1].

Taken all together, we uncovered a novel nuclear role for *Drosophila* Atg8a which is governed by its ability to bind a LIR motif-containing transcription factor, and its acetylation status, and determines its nuclear localization and the regulation of expression of autophagy-related genes (Figure 1). This brings into the spotlight the unanticipated role of a non-degradative LIR motif-dependent interaction in the nucleus, which functions to control cellular self-eating in the cytoplasm.
